# HEPATITIS VIRUS C INFECTION, ADIPOKINES AND HEPATIC STEATO–FIBROSIS

**Published:** 2008-02-25

**Authors:** C Ciurtin, V Stoica

**Affiliations:** Reumatology and Internal Medicine Clinic, Dr. I. Cantacuzino Clinical Hospital,BucharestRomania

## Abstract

Hepatitis C viral infection is accompanied by various serum alterations that could explain its molecular impact on hepatic structure and metabolic homeostasis. Recently it has been shown that adipocytokines play a pivotal role in development of hepatic steato–fibrosis, different studies giving a support of the hypothesis that the balance of adipocytokine expression is a key regulator for the progression of hepatic steatosis and fibrosis.

The association between insulin resistance and hepatitis C virus genotypes and liver fibrosis stage foreshadowed that virus–induced insulin resistance may be a mechanism for fibrogenesis in chronic hepatitis C virus infection.

The main importance of adipocytokine profile detection consists in the prediction of steatosis induction that has clinical relevance, being associated with advanced fibrosis and hyporesponsiveness to antiviral therapy.

Hepatitis C virus infection represents an issue with major impact on populational health in the entire world, due to its high infectious rate and the consequences of its offences brought to liver and extrahepatic tissue.

The plymorphic manifestations of viral hepatitis C plead for the hypothesis of some extremely complex pathogenic mechanisms, which are variably influenced by standard antiviral treatment. The evolution of present diagnosis concepts is directed towards avoiding potentially invasive maneuvers and also towards possibly putting to good use potential markers which certify the nature of infection and become predictive factors of response to treatment.

High therapeutic costs, as well as the adverse effects that can be quite notable compel to the assay and development of some hypotheses that correlate genetic, biochemical, immune and imaging  traits of the viral infection with the potential seriousness of manifestations and the response to treatment. Although the efficacy of interferon therapy in chronic hepatitis C is proven in numerous clinical trials, there are still many cases of dissatisfactory responses to this therapy [1].

Biochemical alterations that accompany the viral replication are insufficiently known up to this day. Infection with hepatitis C virus encloses a lot of metabolic effects which are correlated with the accumulation of fat in vitro and in vivo, changes in lipidic serum profile along with the presence of a certain degree of fatty liver uptake [2, 3].

Many studies have tried to bring to light the pleiotropic effects of C virus replication in order to establish connections between viral load levels and the presence of various manifestations.

It is presently well known the existing association among the different virus genotypes and the level of fat upload in the liver as well as the various levels of response to the antiviral conventional treatment [3,4, 5].

Different links with lipidic profile alterations have raised a particular interest as they have offered, at least partially, explanations concerning the mechanism of viral operation [6 ,7 ].

The effects of hepatitis C virus on fat metabolism are partially identified. It is consentingly accepted the fact that the C virus induces the accumulation of lipids and that there is clearly a connection between the viral genotype and the level of lipidic upload in the liver. Genotypes Ⅰb, Ⅲa and Ⅲh are connected with the increase of triglycerides, which can give explanations concerning the induction of liver steatosis associated with hepatitis C, as genotype Ⅲ is connected with a ‘viral’ type of steatosis and the other HCV genotypes with a ‘metabolical’ type of steatosis [7, 8].

Other observations on metabolic effects have distinguished the fact that HCV presence goes along with low serum cholesterol and apolipoprotein B levels, and that the response to the antiviral treatment seems to have a reversible result on these alterations of lipidic metabolism [8, 9].

Various explanations have been sought to connect the presence of the virus to the lipidic profile, like the existence of oxidative stress caused by the increase of lipid peroxidation, a mechanism which can be involved in the pathogenesis of hepatitis C [9, 10].

There has even been an elaboration of a hypothesis concerning the connection between the rise of serum cholesterol, during and after interferon therapy, with the activity of serum colinesterase.

The significant alterations that viral replication induces on liver cell architecture, evaluated in standard histological scores of necrotic and inflammatory activity, steatosis and degree of fibrosis, denotes the presence of extremely complex cellular and metabolic signal mechanisms.

In the circumstances of dismetabolic factors absence, well known as inductors of fatty deposits in the liver as well as in the presence of low or normal levels of serum cholesterol (which are frequently correlated with an intense viral replication), the mechanism of  steatosis induction in the liver seems to be completely different than the one in diabetes mellitus or the one in the metabolic disfunctions of other causes [11, 12, 13, 14, 15].

In this aspect, the investigation of serologic markers to be associated with the tendency of lipid accumulation in the liver, in comparison with the cytokines involved in the steatosis of diabetes mellitus, looks extremly stimulating.

The prevalence of hepatic steatosis is established to be increased by obesity and DM, with plain fatty liver or nonspecific inflammatory steatosis having a better long term prognosis, while non–alcoholic steato–hepatitis (NASH) may lead to cirrhosis.

Risk factors for NASH are represented by DM, BMI over 25 kg/m^2^ and central obesity [16, 17,18]. Insulin resistance appears to be the main factor involved in the complex pathogenesis of fatty liver, and its association with the presence of HCV determines a low rate of response to the standard antiviral therapy, although insulin resistance that accompanies HCV infection is incriminated as a risk factor for the development of DM in the carriers of infection [19].

Insulin resistance expressed in hepatocytes constitutes the result of alterations in a group of serologic and local factors such as hyperglicemia, hyperinsulinemia, the formation of advanced glycation compounds, the rise of free fatty acids and their metabolic outputs, the increase of oxidative stress as well as the altered adiponectin profile.

The stellate cells are not only passive witnesses of these processes but also target cells that have the ability to respond to pathogenic alterations that appear simultaneously with the development of insulin resistance.

There are more than one mechanisms that act individually and in combination to determine an inhibiton of the insulin signaling sequence. Fatty tissue represents a key site for the interaction of adipokines with inflammation and immune cells, having a role in the production of adipocytokines. These peptides have an extremely important function in modeling the sensibility to insulin, the lipidic metabolism as well as the immune and inflammatory reactions. Changes in their secretion is most relevant in obesity and in metabolic syndrome [20, 21].

Adiponectin is the most abundant adipokine in plasma and its production decreases in the context of obesity and insulin resistance. It has anti–inflammatory qualities and an effect in the rise of hepatocyte sensitivity to the gluconeogenesis inhibition mediated by insulin. Due to its complex mechanisms of action in hepatocytes, mediated by the adipo R2 receptor, adiponectin fights the lipid uptake within these cells. It is reckoned that liver tissue that is uploaded with fat in the presence of peripheral insulin resistance, is comparable with fat tissue. Liver inflammation and fibrosis can be a consequence of its exposure to metabolic and pro–inflammatory mediators produced by visceral fat and brought at this level through the portal venous system [20,21, 22].

While cytokines like TNF–alfa and IL1 have a profibrotic effect and determine insulin resistance, adipocyte hormones have an opposite result; leptin and visfatin improve insulin resistance, while adiponectin and resistin increase it and have an anti–fibrosis effect. There are also many other molecules which have only partially known effects, like adipsin, PAI–1 and angiotensinogen.

Multiple experiments carried out on animals have shown that leptin is a mediator of fibrogenesis and repair through fibrosis of tissular injuries. There isn't yet a strict correlation between serum concentration of leptin and the level of activation of its hepatocyte receptor, where there can be induced a local production or/and an increased  accumulation of leptin. Although steatosis and fibrosis have insufficiently elucidated mechanisms, they seem to be pathogenically independent of leptin, even though once the process has started, leptin determines an acceleration of fat uptake inside the hepatocytes, a process that is dependent also on the grade of the patient's insulin resistance. In the future, it is considered that by selectively blocking leptin signaling in the stellate liver cells, an optimal therapy aimed against hepatic fibrosis may be developed.

Also, free fatty acids and activation of inflammatory paths are other determining factors for the progression of lesions to cirrhosis. Other molecular structures involved directly in fibrotic regeneration are plasminogen activator inhibitor 1, chemotactic monocyte protein 1 as well as some cytokines with favourable effect on inflammation (TNF alfa, IL6) and fibrosis (TGF–beta) [25, 26](Table 1).

**Table 1:** 

Subtype	Adipocitokine	Effect on hepatocyte	Effect on stellate hepatic cell	Effect on Kupffer cells	Effect on endothelial sinusoidal cells
Cytokine	TNF alpha	Insulin resistance	Anti–fibrogenessis or pro–fibrogenessis	Activation	Unknown
	IL6	Insulin resistance (SOC 3 dependent)	pro–fibrogenessis	Activation	Protection against apostosis
Chemokine	MCP–1	Unknown	Migration stimulation	Recruitment	Unknown
Growth factors	VEGF	Unknown	Enzymatic phosphorylation	Unknown	Stimulation of proliferation and increase of permeability
Hormones	Leptin	Improves insulin resistance	Pro–fibrogenic	Increases the effect of TGF beta	Increases the effect of TGF beta
	Adipo Nectin	Increases sensitivity to insulin	Anti–fibrogenic	Anti–inflammatory	Unknown
	Resistin	Insulin resistance	Unknown	Unknown	Unknown
	ASP	Unknown	Unknown	Unknown	Unknown
	Visfatin	Improves insulin resistance	Unknown	Unknown	Unknown
	Adipsin	Unknown	Unknown	Unknown	Unknown
Vasoactive peptides	Angiotensinogen	Unknown	Profibrogenic (converted to angiotensin II)	Pro–inflammatory(converted to angiotensin II)	Unknown
Inhibitor de fibrinoliza	PAI–1	Unknown	Pro–fibrogenic	Unknown	Unknown

The increased expression of cytokine signaling, SOCS–1 and SOCS–3, is involved in the pathogenesis of metabolic syndrome and insulin resistance by means of their ability to simultaneously modulate insulin and cytokine signals alike [26, 27, 28].

Adipokines, SOSC–3 and hyperinsulinemia have been nominated as determining factors of the extent of fibrosis and of the unresponsiveness to pegylated interferon (PEG IFN), the result and duration of the antiviral treatment depending on the patient metabolic and nutritional status and on viral genotype.

The increased expression of SOCS–3, induced by the obesity related to insulin resistance, through a rise in adipocyte secretion of TNF– alpha and leptin, appears to be one of the inhibiting factors of PEG IFN induced signaling path through the lowering of nuclear factor STAT phosphorylation [29, 30, 31]

The independent and inverse predictive role of adiponectin concerning the response to the antiviral therapy in hepatitis C is to be mentioned. Also, it is suggested that leptin may be an independently predicitve factor of unresponsiveness to PEG IFN although the pathogenic mechanism is incompletely discovered. The induction of hepatic expression of SOCS–3 may constitute an explanation. Recent studies have shown that patients with high levels of TNF–alpha had a poor response to viral therapy.

As far as the ethiopathogenesis of steatosis in chronic viral hepatitis C goes, recent literature data indicate the involvment of the same mediators of metabolic syndrome, but also of the factors assosciated with oxidative stress, of the lipid peroxidation products as well as the role of hepatocyte apoptosis and of stellate cell activation with the increased deposit of the extracellular matrix elements [32, 33].

In cohorts with genotype Ib HCV, which is predominant in our country, liver steatosis has been linked with metabolic factors that also had a negative impact on sustained response to antiviral treatment [34, 35].

HCV infection induces not only steatosis and increase in TNF–alfa serum levels but also the development of insulin resistance, the presence of steatosis being correlated with that of serum inflammation markers [36].

Taking into account the literature data, the comparative assesment of pro–fibrosis inflammation markers and of adipocyte cyotkines appears to have a great importance in establishing a parallel between the mechanism of steatosis induction in chronic hepatitis C and in DM.
